# Development and evaluation of an intervention to promote the use of eyeglasses among Romani families in Bulgaria

**DOI:** 10.3389/fpubh.2023.1096322

**Published:** 2023-01-24

**Authors:** Gergana Damianova Kodjebacheva, Slavka Grigorova Hristova, Ventsislav Savov

**Affiliations:** ^1^Department of Public Health and Health Sciences, College of Health Sciences, University of Michigan—Flint, Flint, MI, United States; ^2^International Institute, University of Michigan—Ann Arbor, Ann Arbor, MI, United States; ^3^Department of Economics and Management, College of Management, Trade, and Marketing, Sofia, Bulgaria

**Keywords:** eyeglasses, refractive errors, children, families, Roma, Bulgaria

## Abstract

**Objective:**

Uncorrected refractive error (i.e., lack of eyeglasses for the treatment of refractive error) is one of the leading causes of visual impairment in Eastern Europe. Limited information is available on how to promote the use of eyeglasses among Romani families in Bulgaria. In step 1, the objective was to obtain suggestions by Romani mothers on how to promote the use of eyeglasses among children. In step 2, the objective was to evaluate an intervention to promote the use of eyeglasses based on suggestions received during step 1.

**Methods:**

During step 1, 5 focus groups with Romani mothers took place in one neighborhood in Bulgaria. During step 2, the intervention used a one-group pre-test, post-test design. Families received eye examinations. Those who needed eyeglasses chose attractive eyeglasses. Parents received education on how to encourage their children to wear eyeglasses.

**Results:**

During step 1, 54 mothers participated. Mothers suggested that the whole family should receive eye examinations and eyeglasses. During step 2, of 33 family members, 14 did not have refractive errors and 19 did. Of the 19 family members with refractive error, none had eyeglasses at pre-test. Approximately 6 months following the end of the intervention, 11 of the 19 family members (57.9%) wore eyeglasses and the remaining 8 (42.1%) did not.

**Conclusion:**

Romani family members needed eyeglasses but did not have any at pre-test of the intervention. Future interventions that offer education on the importance of eye examinations may increase receipt of eye examinations and adherence to wearing eyeglasses.

## 1. Introduction

In Europe, “Roma” and “Romani” are terms to describe people who self-identify as Roma, Gypsy, Sinti, Travelers, Ashkali, Manush, Dom, and Lom (1). The Roma represent one of the largest and most vulnerable minority groups in Europe (2, 3). The number of Roma is difficult to quantify (3). According to estimates, 10–12 million Roma reside in Europe (4). Six million Roma out of these 10–12 million reside in the European Union (4). Countries with largest populations of Roma in the European Union include Bulgaria, Romania, Slovakia, Hungary, Greece, Czechia, and Spain (4). According to estimates, approximately 784,041 Romani people revised in Bulgaria in 2020 representing 11.7% of the Bulgarian population (5).

The Roma have suffered racism, discrimination, and social exclusion (1). The Roma were the victims of horrific treatment that included slavery and genocide. According to estimates, 5 million Roma were murdered during the Holocaust (1). The Roma were once nomadic; today, the Roma have varied residences within nomadic, semi-nomadic, and settled groups (6). Lags in education among the Roma and existence of discrimination contributed to high unemployment and access to primarily low skilled jobs among the Roma (7).

The overall health of the Romani population is worse than that of the general population due to factors such as extreme poverty, high unemployment, domestic violence, alcoholism, and malnutrition (8–10). Infant mortality and decreased socio-economic status contribute to the lower life expectancy of the Romani population when compared to the general European population (3). Researchers emphasized that the wide gap in income that exists between Roma and non-Roma across Europe should be reduced as a priority, through targeted interventions (3, 10, 11).

Romani children suffer from worse health outcomes compared to other children in Europe. Roma infants have increased odds of having low birthweight and birth defects (12–15). The low access to safe sex education and reproductive health services results in teenage and unwanted pregnancies (16, 17). Roma children have higher rates of communicable diseases than other children (18). A combination of social isolation, lack of education, domestic violence, and absence of community health programs leaves Romani families including children severely disadvantaged (8, 10, 11).

While studies on the general health of Romani children have been conducted (12–15, 19, 20), research on eye health and care is very limited. Uncorrected refractive error is defined as the lack of eyeglasses for the treatment of myopia, hyperopia, and astigmatism (21–24). The lack of eyeglasses among children with vision problems may result in decreased academic achievement (25, 26). Even in schools with higher socio-economic status in the United States, over 95% of first-graders who needed eyeglasses did not have eyeglasses and/or did not wear them (21). In adults, uncorrected refractive error was associated with reduced vision-related quality of life (27). Research identified uncorrected refractive error as one of the leading causes of correctable visual impairment in Eastern Europe (28–30). Limited information is available on the eye care of Roma children in Eastern Europe. A study of adults in Hungary published in 2022 found that in groups with visual acuity below 0.5 in both eyes, the percentage of people wearing eyeglasses was significantly lower in Roma compared to non-Roma (14.3 vs. 77.1%, *p* < 0.001) (31).

Given the gaps in the research, the current study consisted of 2 steps. During step 1, the study focused on understanding the eye care needs of Romani children by conducting focus groups with mothers. Step 1 sought to receive suggestions for strategies that promoted the use of eyeglasses among Romani children by Romani mothers from one poor Romani neighborhood in Bulgaria.

During step 2, the study implemented an intervention to increase the use of eyeglasses among Romani families in the same poor Romani neighborhood in Bulgaria by using suggestions provided by mothers during step 1. During step 2, the study tested the feasibility of an intervention that offered complimentary eye examinations and attractive eyeglasses to family members and provided education to parents on how to encourage children to wear eyeglasses. During step 2, the study tested the effectiveness of the intervention in promoting the use of eyeglasses by using 2 methods: (1) randomly visiting the neighborhood to observe if family members wore their eyeglasses and (2) conducting focus groups with parents to receive feedback on the use of eyeglasses. Step 2 allowed to investigate what proportion of those who needed eyeglasses based on the optometrist examination had eyeglasses at pre-test.

## 2. Methods

### 2.1. Steps, ethical approval, and setting

The study consisted of 2 steps (Supplementary Figure 1). We received Institutional Review Board (IRB) approval from the University of Michigan—Flint for both steps. The IRB categorized step 1 as exempt due to the limited risk for the focus group participants. The IRB categorized step 2 as a “no more than minimal risk study” since the use of eyeglasses was accepted and common in society.

Suggestions that participants provided during the focus groups during step 1 were used to develop the intervention during step 2. Recruitment for step 2 began ~13 months after the end of the last focus group during step 1. The 13 months were needed to analyze the focus group information during step 1, develop the intervention for step 2, receive IRB approval for step 2, and partner with a local optometrist for step 2. Recruitment for steps 1 and 2, therefore, was completed independently. Verbal informed consent for participation was required for step 1 of the study. Written informed consent for participation was required for steps 2 of the study. The consent forms were verbally described and/or read as needed. It was emphasized that participation was voluntary.

Both steps 1 and 2 were conducted in Bulgarian in Bulgaria meaning that the consent forms were provided to participants in Bulgarian and all focus groups and activities were conducted in Bulgarian. All study materials such as consent forms and focus group guides were submitted to and approved by the IRB in English with translations in Bulgarian.

The setting for both steps 1 and 2 was a neighborhood located in the outskirts of the city following an unpaved, steep, and curvy road. No public bus traveled to the neighborhood. The study team visited the neighborhood by car while driving slowly due to the road condition. Neighborhood residents could be seen walking on foot going to and from the neighborhood. No stores, pharmacies, or other businesses were located in the neighborhood. Farm animals could be seen outside in front of the houses. Children playing and adults interacting could be observed in the neighborhood.

### 2.2. Step 1: Focus groups among mothers

Five focus groups with Romani mothers in one neighborhood where Romani people concentrated in one industrialized city in Bulgaria were conducted in Bulgarian to (1) understand mothers' experiences with any prior vision screening among children, issues surrounding wearing eyeglasses among children (such as factors preventing children from wearing eyeglasses, mother's perceptions toward children wearing eyeglasses, mothers' perceptions on what types of eyeglasses looked better than others, and peer bullying among children due to wearing eyeglasses), cultural perceptions toward wearing eyeglasses, knowledge on the benefits of eyeglasses and eye conditions in general, and perceptions on the importance of eyesight and (2) recommend strategies for the increased receipt and use of eyeglasses among Romani children. Focus groups are established research techniques to explore attitudes, opinions and perceptions through the use of open-ended questions (32).

#### 2.2.1. Inclusion criteria

The inclusion criteria were: Each mother had to understand and speak Bulgarian and have the responsibility for taking care of at least one child (aged 5–17 years) to be eligible. Biological mothers, stepmothers, and guardians (referred to as “mothers” in this study) were eligible to participate. The numbers of mothers for each focus group were: 11 mothers in focus group 1, 9 in focus group 2, 12 in focus group 3, 10 in focus group 4, and 12 mothers in focus group 5.

#### 2.2.2. Recruitment of mothers

In the Romani neighborhood, people spend much of their time outside. When outside visitors arrive in the Romani neighborhood, Roma gather outside of their homes to greet the visitors. The five focus groups took place during five visits to the neighborhood. As Roma people gathered outside of their homes, they were invited to participate in the focus groups if they met the inclusion criteria. As residents were approached, residents stated they would bring additional mothers who may want to join. The focus groups took place shortly after mothers expressing interest gathered and after the consent forms were described and provided to mothers, any questions were answered, and verbal consent was received. It was emphasized that participation was voluntary. The focus groups took place outside, in front of houses in the neighborhood where places for seating were available. Refreshments were provided for both mothers and accompanying children.

A focus group guide (Table 1) was developed by the author (GK) who had previous experience in conducting focus groups among parents on the need and use of eyeglasses in the United States (33). While mothers had to have at least 1 child aged 5–17 to participate, mothers were asked regarding issues surround eye care for any of their children aged 5–17 years. Each focus group took between 1 and 1.5 h to conduct. The focus group discussions were categorized under common topics/themes using grounded theory techniques (32).

**Table 1:** Focus group guide questions^*^ asked during step 1 of the study.

**Topic/question**
**Vision screening in the office of the pediatrician**
When do you recall that the eyes of your child were screened for the first time ever, if ever? Where? How?
Describe your experience with children's vision screening at the office of the pediatrician?
How does the pediatrician screen the eyes of the child?
What happened after the vision screening at the pediatrician's office?
How would you (did you) feel if the pediatrician told you your child might need eyeglasses?
**Issues surrounding wearing eyeglasses**
Do your children wear eyeglasses? For what conditions?
How do you feel (would you feel) about your child (if your child wore) wearing eyeglasses?
How do you think children feel about wearing eyeglasses?
What prevents children from wearing their eyeglasses?
Do some eyeglasses look better than others? Please explain.
What would you do if your child does not want to wear his/her eyeglasses?
Have you heard of negative experiences in children when children wear eyeglasses?
**Cultural perceptions**
What is the general attitude toward wearing eyeglasses among people around you?
What do your relatives and you think about others who wear eyeglasses?
Are there any names relatives use for people wearing eyeglasses?
**Knowledge on the benefits of eyeglasses and on eye conditions in general**
Do you think eyeglasses help children? How?
Do you think eyeglasses hurt children? How?
Is not wearing eyeglasses a serious problem in the development of the child? How?
What are the consequences of not wearing eyeglasses in children?
What eye conditions have you heard of?
**Perceptions on the importance of eyesight**
How important are people's eyes to their health and wellbeing?
**Strategies for the increased receipt and use of eyeglasses among Romani children**
What do you suggest be done so that we improve children's eye care?
What do you suggest be done to evaluate/examine the eyes of the children?
What do you suggest be done so that children have eyeglasses in they need eyeglasses?
What do you suggest be done to help children wear their eyeglasses if they need eyeglasses?

* While mothers had to have at least 1 child aged 5–17, mothers could discuss issues related to any of their children aged 5–17 when answering questions.

### 2.3. Step 2: Intervention to promote the use of eyeglasses among family members

#### 2.3.1. Inclusion criteria

Children aged 5–17 years residing in the Romani neighborhood where the focus groups in step 1 were conducted were the most important part of the target population. All children within these ages from the same family were eligible.

During the focus groups in step 1, mothers stated that the family (i.e., mothers and fathers along with children) should have the opportunity to visit the optometrist. Mothers stated that if they and fathers wore eyeglasses, their children would be encouraged to wear them as well. We realized that because Roma people were part of an impoverished community, all family members (i.e., mothers, stepmothers, or female guardians and biological fathers, stepfathers, or male guardians along with children aged 5–17) should be given the opportunity to visit the optometrist and receive free eyeglasses. Children's parents were thus also eligible to participate in step 2.

The inclusion criteria for inclusion in the intervention thus were children aged 5–17 years and their parents such as biological mothers, stepmothers, or female guardians and/or biological fathers, stepfathers, or male guardians. A family was defined as the children aged 5–17 years and the parents who could be biological mothers, stepmothers, or female guardians and/or biological fathers, stepfathers, or male guardians. Grandparents were not eligible to participate unless they were guardians. From this point on, mothers, stepmothers, and female guardians are called mothers. Biological fathers, stepfathers, and male guardians are called fathers. Parents were not required to have eye examinations; they could choose to either have eye examinations only for their children and/or for both them and their children. Parents could not choose to have eye examinations only for themselves.

#### 2.3.2. Recruitment of adults (i.e., mothers and fathers) and children

The intervention was advertised by approaching residents in the same neighborhood as in step 1. As Roma people gathered outside of their homes, the intervention was verbally described.

The consent form was developed in English and translated into Bulgarian. Some adult participants were illiterate. The consent form was both read/explained and provided to the adults. Participants were asked if they had questions. It was emphasized that participation was voluntary. Adults asked their children to participate. The following was stated in the form: “Please ask your child or the child you care for aged 5–17 years if he/she would like to participate in this research. If the child does not wish to participate, you do not need to come to the office of the optometrist. The child is free to refuse participation in the study. If you have or care for more than 1 child within these ages, you can ask each child to participate.”

#### 2.3.3. Intervention design

A one group pre-test post-test intervention was conducted (Supplementary Figure 1).

#### 2.3.4. Intervention strategies

The intervention strategies were:

- Complimentary eye examinations by an optometrist in the office of the optometrist for children and their mothers and/or fathers. Free transportation to the office of the optometrist was not provided. Participants went to the office of the optometrist on their own. The optometrist office was located close to the main hospital in the city. The office was open Monday–Friday 8 a.m.−7 p.m. and Saturday 8 a.m.−1 p.m. The optometrist was available to offer eye examinations only on Mondays and Wednesdays 9 am – 6 pm.
- Provision of complimentary eyeglasses that participants selected at the office of the optometrist. Provision of eyeglasses was based on the clinical decision of the optometrist according to the refractive error status of the patient. The office of the optometrist was reimbursed for the eye examinations and eyeglasses by the study grant.
- Eye care education for mothers and fathers on how to encourage children to wear eyeglasses. The education was provided by the optometrist during the visit using strategies discussed between the study team and the optometrist. To encourage children to wear eyeglasses, parents were advised to allow children to participate in choosing frames, take slow steps while children become used to their eyeglasses without forcing children to wear the eyeglasses, practice taking eyeglasses on and off, and return to the office of the optometrist if eyeglasses needed adjustments.

#### 2.3.5. Quantitative outcome evaluation with the outcome measure use of eyeglasses at pre- and post-test

To assess if the intervention was effective, the optometrist asked participants if they had eyeglasses during the eye examination at the start of the intervention; the numbers of participants who needed eyeglasses and who already had eyeglasses were recorded at pre-test. The neighborhood was then randomly visited a total of 3 times to count the number of people wearing their eyeglasses to understand if the intervention was effective ~6 months after the provision of the eyeglasses at post-test. The neighborhood was visited 3 times in case some of the participants were not in the neighborhood at the time of the visit. Use of eyeglasses was measured only at the first visit when the participant was encountered. If participants had to wear their eyeglasses only for close work according to the optometrist notes, participants were asked if they wore their eyeglasses while doing close work and were asked to bring their eyeglasses; if participants brought their eyeglasses, they were marked as wearing their eyeglasses. If participants had to wear their eyeglasses all the time according to the notes of the optometrist and if participants did not wear the eyeglasses at the time of the visit, the participants were marked as not wearing their eyeglasses.

#### 2.3.6. Qualitative outcome and process evaluations: Focus groups at post-test only

During the 3 random visits to check if participants wore their eyeglasses ~6 months after the receipt of eyeglasses, we sought to conduct focus groups with the mothers and fathers who received eyeglasses either for themselves and/or their children to obtain information on how wearing eyeglasses may have affected participants and suggestions for improving the intervention. Children were excluded from the focus groups. All adults who received eyeglasses for themselves and/or their children regardless of whether they and/or their children adhered to wearing them were invited to participate in the focus groups during step 2.

The focus groups during step 2 served as both outcome and process evaluations. The outcome evaluation focused on how the intervention may have improved family members' vision and lives. The process evaluation focused on suggestions adults had to improve the intervention. During the first random visit, participants stated they were not available for a focus group due to lack of time. During the second random visit, one focus group was conducted and during the third random visit, a second focus group was conducted. A focus group guide was used. Questions asked during the step 2 focus groups included:

*Outcome evaluation to assess adherence to wearing eyeglasses and influence of using eyeglasses on life:*

- How frequently did you/your child(ren) wear the eyeglasses?
  ° Why did you wear them frequently/less frequently?
  ° Where did you/your child(ren) wear them more frequently (at home, in school, or outside)?
- How did your life (the life of your child or children) change after receiving the eyeglasses?

*Process evaluation to receive suggestions for improving the intervention:*

- What was your overall experience with the program?
- What was your overall experience at the office of the optometrist?
- Please share any problems you had during this program.
- What suggestions do you have to improve the program in the future?

## 3. Results

### 3.1. Step 1: Focus groups among mothers

#### 3.1.1. Mother's characteristics

The 54 mothers were either unemployed or had part-time employment (Table 2). All mothers had either completed no education or completed one to four grades. Seventy-five point nine percent of mothers (41) had 4 children younger than 18.

**Table 2:** Characteristics of mothers participating in the five focus groups during step 1, *n* = 54.

	**Number**	**%**
**Role**		
Biological mother	41	75.9%
Stepmother	7	13.0%
Guardian	6	11.1%
**Age**		
18–29	6	11.1%
30–39	16	29.6%
40–49	21	38.9%
50–67	11	20.4%
**Education**		
No formal education	12	22.2
First-grade education	15	27.8
Third-grade education	18	33.3
Fourth-grade education	9	16.7
**Employment**		
Unemployed	9	16.7
Employed part-time	45	83.3
**Number of children younger than 18**		
2	6	11.1
3	13	24.1
4	22	40.7
5	13	24.1

#### 3.1.2. Vision screening for children

The mothers stated that their children aged 5–17 had received vision screening by the pediatrician. The parents described the vision screening. The pediatrician used an eye chart. The pediatrician then referred the child to the optometrist if visual impairment was suspected. The parents felt respected by the pediatrician. The pediatrician answered parents' questions. No mother stated to have been to the optometrist.

#### 3.1.3. Eyeglasses

Mothers reported that no child or adult in the neighborhood wore eyeglasses. Some mothers stated that their children needed eyeglasses but did not have any. Most mothers stated that their children had no complaints about their eyes and did not need eyeglasses. Mothers stated that they could not afford to purchase eyeglasses for their children. Some of the mothers stated that they needed eyeglasses for themselves but could not afford them. Some mothers asked if the moderators could purchase eyeglasses for them. The mothers stated that they found some frames for eyeglasses more attractive than others; they preferred thinner frames.

#### 3.1.4. Mother's perceptions and knowledge related to eyesight and vision

The mothers stated that they understood the importance of wearing eyeglasses. One mother stated: “Eyes carry the future.” Mothers stated that eyes along with the head and legs were the most important body parts. They stated that if one had a problem with his/her eyes, the problem would be serious because eyes were a part of the head. When asked what the benefits of eyeglasses were, the mothers stated that eyeglasses helped children see well and do better in school. Mothers believed that if children had a problem with their eyes, they would be able to communicate the problem with the parents. Mothers stated that they had no objections to children wearing eyeglasses. If their children needed and received eyeglasses, the mothers would encourage and even require the children to wear eyeglasses. The mothers stated that they were unaware of cases of bullying among the children due to wearing eyeglasses. Mothers had heard of farsightedness and nearsightedness. They could not appropriately define the conditions. Mothers had heard of cataracts; several parents stated their relatives were diagnosed with and treated for cataracts.

#### 3.1.5. Suggestions on promoting the use of eyeglasses

Mothers stated that the whole family (mothers, fathers, and children) should visit the optometrist. The whole family should receive complimentary eye examinations and eyeglasses if needed. The mothers would like to receive education on how to encourage their children to wear eyeglasses.

### 3.2. Step 2: Intervention to promote the use of eyeglasses among family members

#### 3.2.1. Characteristics of intervention participants

A total of 33 family members visited the office of the optometrist. Among the 33 participants, 75.8% (or 25) were female and 69.7% (23) were aged 5–17 years (Table 3). The 33 participants represented a total of 14 families.

**Table 3:** Age and gender of the 33 participants whose eyes were examined by the optometrist during step 2 of the study.

**Characteristic**	**Number**	**%**
**Age**		
5–9	4	12.1%
10–14	9	27.3%
15–17	10	30.3%
18–29	2	6.1%
30–39	1	3.0%
40–49	1	3.0%
50–67	6	18.2%
**Gender**		
Male	8	24.2
Female	25	75.8

#### 3.2.2. Feasibility of the intervention

One of the authors (SGH) was present in the waiting room at the office of the optometrist for all scheduled eye examinations. Romani people seemed enthusiastic to receive eyeglasses. Roma selected eyeglasses that they stated they liked.

#### 3.2.3. Quantitative outcome evaluation with the outcome measure use of eyeglasses at pre- and post-test

Out of the 33 family members, 14 did not have refractive errors and 19 had refractive errors according to the results of the examination by the optometrist. Of the 19 family members with refractive error, none previously had eyeglasses at pre-test (Table 4). Approximately 6 months following the end of the intervention at post-test, 11 of the 19 family members (57.9%) wore eyeglasses and the remaining 8 (42.1%) did not. The following are the age groups of the 19 participants with refractive error who received eyeglasses by adherence to the use of eyeglasses at post-test. Among the 11 who adhered to wearing eyeglasses at post-test, 2 were aged 10–14, 4 were aged 15–17, 1 was aged 18–29, 1 was aged 40–49, and 3 were aged 50–67. Among the eight who did not adhere to wearing eyeglasses at post-test, 2 were aged 5–9, 2 were aged 10–14, 1 was aged 15–17, 1 was aged 18–29, and 2 were aged 50–67. At post-test, out of the eight people who did not have/wear their eyeglasses, two broke the eyeglasses, four expressed that the eyeglasses were not comfortable, and two expressed that there was no need for eyeglasses due to good vision.

**Table 4:** Children with and without eyeglasses at the beginning of the intervention (i.e., during the appointment with the optometrist) and ~6 months after the provision of eyeglasses among the 19 participants who needed eyeglasses during step 2 of the study.

**Use of eyeglasses**	**Yes**		**No**	
	**Number**	**%**	**Number**	**%**
Wore eyeglasses at the beginning of the intervention	0	0	19	100
Wore eyeglasses ~6 months after receiving them	11	57.9	8	42.1

#### 3.2.4. Qualitative outcome and process evaluations: Focus groups at post-test only

Out of the 19 parents who received eyeglasses, six agreed to participate in the focus groups. Out of the six parents who agreed to participate in the focus groups, five adhered to wearing eyeglasses (either for themselves and/or their children) and one did not adhere due to accidentally breaking the eyeglasses. These six parents represented six families. All six parents who participated in the focus groups were mothers. Mothers expressed they were very thankful for the services offered and did not express problems. As part of the outcome evaluation to understand how wearing eyeglasses affected people's lives, mothers stated that they and/or their children could see better as a result of receiving eyeglasses. Some participants stated that they preferred to only wear their eyeglasses at home and did not like to be seen with them outside. Mothers stated they would continue to wear their eyeglasses especially at home. One mother expressed gratitude for the program with tears in her eyes stating that she could not do close work such as knitting prior to receiving eyeglasses. As part of the process evaluation to receive suggestions on improving the intervention, we asked follow-up questions so that mothers would freely express any problems and satisfaction with the intervention, but they continued affirming that they experienced no problems and had no recommendations. One mother who not adherent to wearing her eyeglasses stated she broke the eyeglasses and requested to receive new eyeglasses.

## 4. Discussion

### 4.1. Summary

#### 4.1.1. Step 1

No child was reported to wear eyeglasses in the poor Roma neighborhood. Some mothers stated that they needed eyeglasses or had problems with their eyesight. Mothers could not afford to purchase eyeglasses for themselves or their children. Mothers recognized the positive influence of eyeglasses for the development of children.

Valuable lessons were learned regarding the feasibility of conducting focus groups in the poor Roma neighborhood based on step 1. All focus groups took place in front of the homes of Roma people as requested by the Roma. There were distractions from people passing and by children playing. In the future, it will be useful to locate quieter places for focus groups in close proximity to the Romani neighborhood. Recruitment was a smooth process. Roma were very friendly and wished to participate.

#### 4.1.2. Step 2

The results indicated that Roma in the neighborhood in this study needed eyeglasses but did not have any at pre-test. The intervention increased the use of eyeglasses even months after providing the eye care. A relatively large percentage of all participants who visited the office of the optometrist received eyeglasses (19 out of 33 or 57.6%); the large percentage may be because people who experienced problems with their eyesight were motivated to visit the optometrist.

Lessons were learned regarding the feasibility of conducting the intervention. Initial recruitment was a smooth process. Roma were friendly and wished to participate in the intervention in the beginning. However, initial agreement to participate did not mean that Roma would attend the office of the optometrist. On several occasions, the author (SGH) waited for participants at the office of the optometrist at the arranged time, but none arrived. Lack of time and transportation barriers may have been reasons why participants who initially agreed did not attend the office of the optometrist. Lack of education on the need for eye examinations prior to the visits to the office of the optometrist may have contributed to Roma not wishing to visit the optometrist. Another plausible explanation is that the people who did not attend the office of the optometrist may have not perceived they had vision problems. Males were especially less likely to participate than females. Past research found that males were more likely to ignore vision symptoms and less likely to seek early care compared to females (34). A hypothesis why males have a decreased likelihood to seek preventative health services is related to masculine gender norms where seeking help is considered a sign of weakness among males (35, 36). Another hypothesis for the decreased likelihood to seek preventative health is alcohol and substance abuse among some males (35). Other issues during the intervention included insistent requests by Roma to be paid for their participation and receive payment to cover their monthly utility bills. Roma were informed that the goals of the intervention were to offer eye examinations and eyeglasses.

### 4.2. Limitations

#### 4.2.1. Step 1

The total number of mothers in the neighborhood was not ascertained; therefore, the percent of mothers in the neighborhood who participated was not known. The sample size of participants was small. Romani participants were not invited to a separate location where the focus groups could take place. Instead, focus groups took place outside in the neighborhood. At the same time, given transportation and time barriers, conducting the focus groups where participants were may have contributed to the larger sample size for each focus group.

Mothers stated during the focus groups that the pediatrician had referred the child to the optometrist if visual impairment was suspected. No mother, however, stated to have been to the optometrist. This study did not elaborate on the reasons why mothers did not visit the optometrist despite having referrals. Yet another limitation is that the findings of the focus groups may not be generalized to all Roma mothers/children in Bulgaria. The opinions of fathers were not taken into account since fathers did not participate in the focus groups in step 1. It is not known how many children of mothers participating in the focus groups may have needed eyeglasses.

#### 4.2.2. Step 2

The number of participants who were invited while visiting the neighborhood was not ascertained. Another limitation of the intervention is that the sample size of participants was small. Another limitation is that the percentage of male participants was low. Yet another limitation is that the findings may not be generalized to all Romani parents/children in Bulgaria. Transportation was not offered to the office of the optometrist. Both children and parents participated. Parent could choose to have eye examinations only for the children. Another limitation is that the study did not investigate the prevalence of refractive error and type of refractive error. Provision of eyeglasses was based on the assessment of the optometrist. It is likely that individuals with worse refractive error were more likely to wear their eyeglasses due to poor vision.

Another limitation is that it is uncertain whether participants wore their eyeglasses regularly. On random visits 6 months after the end of the intervention, some participants may have put on their eyeglasses only because they heard that the authors were visiting the neighborhood. Therefore, the random visits were supplemented with the use of focus groups. A study limitation is that the participants who initially agreed to participate in the intervention and visit the optometrists were not interviewed to understand their reasons for lack of participation. Five out of the six participants in the focus groups in step 2 adhered to wearing eyeglasses (either for themselves and/or their children). Having additional participants who lacked adherence to wearing eyeglasses either for themselves and/or their children in the focus groups could have resulted in more recommendations on how to improve the intervention.

### 4.3. Implications

In the literature, up to 30% of children aged 2-17 overall may need eyeglasses (22, 23). In 2019, in the United States while there were still very high rates of uncorrected refractive error, according to the CDC, among boys, 3.0% wore eyeglasses among those aged 2–5 years, 20.0% among those aged 6–11 years, and 35.3% among those aged 12–17 years (37). Among girls, the respective percentages were 3.1, 26.4, and 48.2% (37). The Romani mothers stated during the step 1 focus groups that no one in the neighborhood wore eyeglasses which may be due to financial and other barriers. During step 2, none of the individuals who needed eyeglasses had them at pre-test. An intervention is needed to offer eye examinations by optometrists in the Romani neighborhood to understand what percentage of Romani children need eyeglasses. Future interventions should offer eye care education to parents prior to visiting the optometrist to increase the likelihood that parents understand the importance of eye care visits.

The current intervention in step 2 needs to be enhanced to increase the use of eyeglasses. Initial and follow-up visits by optometrists in the neighborhood to provide, adjust, and/or replace eyeglasses will be valuable. Future focus groups or interviews may ask parents what strategies they recommend to encourage the use of eyeglasses in school. Partnering with schools to assist children in wearing their eyeglasses may be especially effective. Teachers should be informed when children need eyeglasses. Children can receive a second pair of eyeglasses to wear in school such as a in a previous intervention among non-Romani children (24). Teachers can remind children to wear their eyeglasses in school (24). Such a program that involves a partnership with schools may promote the academic development of children should be developed with the suggestions by Romani parents and even children.

## 5. Conclusion

Based on the suggestions by mothers during the step 1 focus groups, future interventions may include: complimentary eye examinations by an optometrist for children and parents in the neighborhood; provision of complimentary eyeglasses; and eye care education for parents on the importance of eye examinations and how to encourage children to wear eyeglasses both prior to and during/after eye examination visits. Future studies may assess whether such interventions promote the academic and healthy development of Romani children.

Nineteen Romani family members who agreed to participate in our study in one neighborhood in this study needed eyeglasses but did not have any at pre-test. The intervention increased the use of eyeglasses even months after providing the eye care. Roma seemed enthusiastic to receive eyeglasses. Roma were able to select eyeglasses that they stated they liked. There were problems with bringing Roma to the office of the optometrist possibly due to transportation and time barriers as well as perceptions for not needing eye examinations. Future interventions that bring the optometrist repeatedly to the neighborhood where Roma reside may result in higher participation rates and increased use of eyeglasses. They may offer eye care education on the importance of eye examinations to parents prior to optometrist visits so that parents will agree to have eye examinations for themselves and their children. The lessons learned can be used in future efforts to implement interventions in Romani communities and help promote the health of underserved populations.

## Data availability statement

The raw data supporting the conclusions of this article will be made available by the authors, without undue reservation.

## Ethics statement

The studies involving human participants were reviewed and approved by the University of Michigan Institutional Review Board. Verbal informed consent for participation was required for step 1 of the study. Written informed consent for participation was required for steps 2 of the study.

## Author contributions

GDK, SGH, and VS: conceived and designed the study, collected the data, and reviewed final paper. GDK and SGH: designed data collection tools. GDK: performed analysis. All authors contributed to the article and approved the submitted version.

## References

[1] Murray C. A minority within a minority? Social justice for traveller and Roma children in ECEC. Eur J Educ. (2012) 47:569–83. 10.1111/ejed.12009

[2] Csata Z Hlatky R Liu AH. How to head count ethnic minorities: validity of census surveys versus other identification strategies. East Euro Polit. (2021) 37:572–92. 10.1080/21599165.2020.1843439

[3] Parekh N Rose T. Health inequalities of the Roma in Europe: a literature review. Cent Eur J Public Health. (2011) 19:139–42. 10.21101/cejph.a3661 22026288

[4] Romani People in the European Union. European Parliament. Available online at: https://multimedia.europarl.europa.eu/en/package/romani-people-in-eu_20901 (accessed November 8, 2022).

[5] Ilieva N Kazakov B. Projection of the Roma population in Bulgaria (2020-2050). In: International Scientific Conference Geobalkanika. Sofia, Bulgaria (2019). 10.18509/GBP.2019.35

[6] Vermeersch P Ram MH. The Roma. In:Rechel B, editor. Minority Rights in Central and Eastern Europe. London: Routledge (2009).

[7] Milcher S Fischer MM. On labour market discrimination against Roma in South East Europe. Pap Reg Sci. (2011) 90:773–88. 10.1111/j.1435-5957.2011.00354.x

[8] Kozubik M van Dijk JP Rac I. Health risks related to domestic violence against Roma women. Int J Environ Res Public Health. (2020) 17:6992. 10.3390/ijerph17196992 32987921 PMC7579367

[9] Masseria C Mladovsky P Hernandez-Quervedo C. The socio-economic determinants of the health status of Roma in comparison with non-Roma in Bulgaria, Hungary, and Romania. Eur J Public Health. (2010) 20:549–54. 10.1093/eurpub/ckq102 20650945

[10] Fernández-Feito A Pesquera-Cabezas R González-Cobo C Prieto-Salceda MD. What do we know about the health of Spanish Roma people and what has been done to improve it? A scoping review. Ethn Health. (2019) 24:224–43. 10.1080/13557858.2017.1315373 28398074

[11] Fésüs G Östlin P McKee M Ádány R. Policies to improve the health and well-being of Roma people: the European experience. Health Policy. (2019) 105:25–32. 10.1016/j.healthpol.2011.12.003 22217864

[12] Hajioff S Mckee M. The health of the Roma people: a review of the published literature. J Epidemiol Community Health. (2000) 54:864–9. 10.1136/jech.54.11.864 11027202 PMC1731574

[13] Rosicova K Geckova MA van Dijk JP Kollarova J Rosic M Groothoff JW. Regional socioeconomic indicators and ethnicity as predictors of regional infant mortality rate in Slovakia. Int J Public Health. (2011) 56:523–31. 10.1007/s00038-010-0199-3 20976517 PMC3174369

[14] Koval' J Mrosková S Schlosserová A. Natality and infant mortality in Roma children in the Prešov region. Med Srod. (2012) 15:92–101.

[15] Mitrut A Tudor S. Bridging the gap for Roma: the effects of an ethnically targeted program on prenatal care and child health. J Public Econ. (2018) 165:114–32. 10.1016/j.jpubeco.2018.07.007

[16] Cintulová LL Radková L. Social aspects of unplanned pregnancies in teenage Roma girls. Kontakt. (2019) 21:106–12. 10.32725/kont.2018.006

[17] Columbini M Rechel B Mayhew S. Access of Roma to sexual and reproductive health services: qualitative findings in Albania, Bulgaria, and Macedonia. Glob Public Health. (2012) 7:522–34. 10.1080/17441692.2011.641990 22175646

[18] Tombat K van Dijk JP. Roma health: an overview of communicable diseases in Eastern and Central Europe. Int J Environ Res Public Health. (2020) 17:7632. 10.3390/ijerph17207632 33092048 PMC7588998

[19] Janevic T. Romani maternal and child health: moving from documenting disparities to testing progress and interventions to achieve equity. Int J Public Health. (2019) 64:981–2. 10.1007/s00038-019-01255-8 31143961

[20] Zeman CL Depken DE Senchina DS. Roma health issues: a review of the literature and discussion. Ethn Health. (2003) 8:223–49. 10.1080/1355785032000136434 14577997

[21] Kodjebacheva G Brown ER Estrada L Yu F Coleman AL. Uncorrected refractive error among first-graders of different racial/ethnic groups in Southern California: results a year after school-mandated vision screening. J Public Health Manag Pract. (2011) 17:499–505. 10.1097/PHH.0b013e3182113891 21964359

[22] Williamson TH Andrews R Dutton GN Murray G Graham N. Assessment of an inner city visual screening programme for preschool children. Br J Ophthalmol. (1995) 79:1068–73. 10.1136/bjo.79.12.1068 8562537 PMC505342

[23] McCathy CA. Uncorrected refractive error. Br J Ophthalmol. (2006) 90:521–2. 10.1136/bjo.2006.090233 16622075 PMC1857030

[24] Kodjebacheva G Maliski S Yu F Oelrich F Coleman AL. Decreasing uncorrected refractive error in the classroom through a multifactorial intervention. J Sch Nurs. (2014) 30:24–30. 10.1177/1059840513486009 23598569

[25] Kimel LS. Lack of follow-up care after failed school vision screenings: an investigation of contributing factors. J Sch Nursing. (2006) 22:156–62. 10.1177/10598405060220030601 16704285

[26] Zaba JN. Social, emotional, and educational consequences of undetected children's vision problems. J Behav Optom. (2001) 12:60–70.

[27] Lou L Yao C Jin Y Perez V Ye J. Global patterns in health burden of uncorrected refractive error. Investig Ophthalmol Vis Sci. (2016) 57:6271–7. 10.1167/iovs.16-20242 27893092

[28] Kocur I Resnikoff S. Visual impairment and blindness in Europe and their prevention. Br J Ophthalmol. (2002) 86:716–22. 10.1136/bjo.86.7.716 12084735 PMC1771203

[29] Bourne RR Jonas JB Flaxman SR. Prevalence and causes of vision loss in high-income countries and in Eastern and Central Europe: 1990-2010. Br J Ophthalmol. (2014) 98:629–38. 10.1136/bjophthalmol-2013-304033 24665132

[30] Bourne RRA Jonas JB Bron AM Cicinelli MV Das A Flaxman SR. Prevalence and causes of vision loss in high-income countries and in Eastern and Central Europe in 2015: magnitude, temporal trends and projections. Br J Ophthalmol. (2018) 102:575–85. 10.1136/bjophthalmol-2017-311258 29545417 PMC5909755

[31] Losonczy G Piko P Klevering BJ Kosa Z Sandor J Adany R. 2022 Low prevalence of spectacle use in the Hungarian Roma population indicates unmet health needs. Sci Rep. (2022) 12:3873. 10.1038/s41598-022-07880-3 35264669 PMC8907268

[32] Krueger R Casey M. Focus Groups: A Practical Guide for Applied Research. 4th edition. Thousand Oaks, CA: Sage publications (2009).

[33] Kodjebacheva G Maliski SL Coleman AL. Use of eyeglasses among children in elementary school: perceptions, behaviors, and interventions discussed by parents, school nurses, and teachers during focus groups. Am J Health Promot. (2015) 29:324–31. 10.4278/ajhp.120315-QUAL-140 24717070

[34] Crabb DP Saunders LJ Edwards L. Cases of advanced visual field loss at referral to glaucoma clinics - more men than women? Ophthalmic Physiol Opt. (2015) 37:82–7. 10.1111/opo.12328 27862125

[35] Novak JR Peak T Gast J Arnell M. Associations between masculine norms and health-care utilization in highly religious, heterosexual men. Am J Mens Health. (2019) 13:1557988319856739. 10.1177/1557988319856739 31184245 PMC6560804

[36] Silvestrini M Chen JA. “It's a sign of weakness”: masculinity and help-seeking behaviors among male veterans accessing posttraumatic stress disorder care. Psychol Trauma. (2022). 10.1037/tra0001382. [Epub ahead of print]. 36201833 PMC11107421

[37] Centers for Disease Control and Prevention. QuickStats: percentage of children aged 2–17 years who wear glasses or contact lenses, by sex and age group—national health interview survey, United States, 2019. MMWR Morb Mortal Wkly Rep. (2021) 70:865. 10.15585/mmwr.mm7023a4 34111062 PMC8191864

