# Supplementary material for: Development and evaluation of an intervention to promote the use of eyeglasses among Romani families in Bulgaria
Source: Front Public Health. 2023 Jan 24;11:1096322. doi: 10.3389/fpubh.2023.1096322 (PMC9902913; doi:10.3389/fpubh.2023.1096322)
Supplement: Supplementary file 1 [file Table_1.DOCX]

Figure 1: Study steps

1. Five focus groups with Romani mothers were conducted to receive suggestions for promoting the use of eyeglasses among children in one Romani neighborhood.

2. Thirteen months after completing step 1, a one-group pre-test post-test intervention was conducted based on suggestions received during step 1 in the same neighborhood. Families including children aged 5 to 17 and parents were eligible to participate. Approximately, six months after the end of the intervention, random visits to the neighborhood took place to observe if participants wore their eyeglasses and focus groups were conducted to understand participants’ use of eyeglasses and satisfaction with the program.

Figure 2: Intervention study design for step 2 of the study

1. Pre-test measurement of the need to use eyeglasses among 33 family members (i.e. children, parents/guardians) from one Roma neighborhood by an optometrist

2. Implementation of the intervention strategies among the 33 family members

3. Post-test measurement of the use of eyeglasses among the same 33 participants by random visits. Focus groups with adults to understand if participants regularly wore their eyeglasses, how children’s vision and academic development changed/improved, and if parents were satisfied with the intervention.
